# The reliability and validity of the Turkish version of the Oxford Elbow Score

**DOI:** 10.1186/s13018-016-0429-3

**Published:** 2016-09-01

**Authors:** Hayri Baran Yosmaoglu, Deha Doğan, Emel Sonmezer

**Affiliations:** 1 Department of Measurement and Evaluation, Faculty of Educational Sciences, University of Ankara, Cebeci, Ankara, Turkey; 2 Department of Physical Therapy and Rehabilitation, Faculty of Health Sciences, Baskent University, Eskisehir Yolu 20, Baglıca, Ankara, Turkey

## Abstract

**Background:**

Objective measures of outcome ensure reliable decisions with regard to treatment planning. Oxford Elbow Score (OES) is one of the common outcome measures used for assessing quality of life of patients with elbow disorders. OES consists of three domains: pain, elbow function and social/psychological. The aim of this study is to test the validity and reliability of the Turkish version of the OES.

**Methods:**

The study’s sample includes 82 patients with elbow problems. The original version of the OES was translated into Turkish using the Isis Outcomes Translation and Linguistic Validation Process. The construct validity of the Turkish version of the OES was tested using a confirmatory factor analysis. For internal consistency, Cronbach’s alpha was calculated. A Pearson correlation and a dependent sample *t* test were utilised for reproducibility of the OES. For convergent validity, the correlation coefficients were calculated between the domains of the OES and Short Form 36 (SF36). An independent sample *t* test was calculated to determine if there was a significant difference between the scores of the participants from the upper and lower groups.

**Results:**

Confirmatory factor analysis (CFA) indicates that the three-factor structure of the OES was confirmed. Most of the fit indices are at the expected level, except for a root mean square error of approximation and an adjusted goodness of fit index. Cronbach’s alpha was calculated as 0.91 for the whole scale. The results showed that there are positive and high correlations between the first and follow-up assessments (*r* = 0.89, *p* < 0.0001). The Turkish OES version and its dimensions have moderate and significant correlations with domains of SF36 in general. The test results indicated that the mean of each item on three domains of the OES was higher for the upper 27 %, and this difference was significant at the 0.01 level.

**Conclusions:**

The Turkish version of the OES is a reliable, valid, reproducible and practical tool. It can be used for patients with elbow disorders and is recommended for clinician use.

## Background

Outcome measures are very important for assessing patients’ functional status and quality of life [1, 2]. They are the result of tests used to objectively determine treatment protocols. In other words, objective measures of outcome ensure reliable treatment decisions. Outcome measures can be achieved either by functional and clinical tests or by questionnaires. Knowing how the patients feel about their own medical condition is very important for the clinicians to make an accurate diagnosis. Questionnaires are effective tools to obtain the patients’ self-assessment of their symptoms [3].

Elbow joint disorders are one the most common orthopaedic problems faced in Turkey, as well as around the world. Therefore, assessing patient reported outcome measures about elbow disorders might guide clinicians during the treatment process [4, 5]. Dowson and colleagues [6] developed the 12-item Oxford Elbow Score (OES) questionnaire to measure the quality of life of patients with disorders of the elbow joint. OES consists of three components: pain, elbow function and social/psychological. Each item is scored from 0 to 4, and higher scores in each component indicate a worse elbow condition. During the development of the OES, construct validity, internal consistency, sensitivity to change, reproducibility, convergent validity and individual item functioning were tested. The results indicate that the original version of the OES is valid, reliable, practical and sensitive to changes that are of clinical importance [6].

Moreover, transcultural adaptation of the OES has been done for many languages [7, 8]; however, its adaptation into the Turkish language has not been done yet. Thus, the purpose of this study is to adapt the OES to the Turkish culture and to test its reliability and validity.

## Methods

This is a scale adaptation study. In this study, the original version of the Oxford Elbow Score was adapted to the Turkish culture. Below, information regarding the methodology of the study is presented.

### Patients

The study sample included 82 patients, 50 (68 %) of whom had a lateral epicondylitis, 18 (22 %) of whom had an elbow fracture and seven (8 %) of whom had a medial epicondylitis. Of the 82 participants, 47 (57 %) were female and 35 (43 %) were male. The age of the participants varied between 19 and 71. The mean of the participants’ ages was 43.60, with a 14.25 standard deviation. The original version of the OES had three factors. Because the number of respondents exceeded the number of items on the questionnaire (12) by at least a factor of three, a sample size of 82 was considered sufficient [9]. Table 1 presents some demographic information about the patients.

**Table 1:** Patient characteristics

Characteristics		*N* %	Total *N*
Gender	Male	35 (43 %)	82
	Female	47 (57 %)	
Age	19–30	23	82
	31–50	33	
	51 and over	26	
Diagnosis	Lateral epicondylitis	56 (68 %)	82
	Elbow fracture	18 (22 %)	
	Medial epicondylitis	7 (8 %)	

Approval was obtained from the Baskent University Clinical Researches Ethic Committee (KA 13/53-2013), and all patients consented to participate in the study.

### Instruments

#### Original version of the Oxford Elbow Score

The Oxford Elbow Score was originally developed and validated by Dawson and colleagues in 2008. The 12-item questionnaire has three components with an eigenvalue <1.0, which explained 75.1 % of the variance. Each component includes four items. The components are pain, elbow function and social/psychological. The factor loadings in each component vary between 0.62 and 0.90. The correlation between the OES domains and Short Form 36 (SF36) domains, Mayo Elbow Performance Score (MEPS) and Disabilities of the Arm, Shoulder and Hand outcome measure (DASH) varies between 0.22 (the minimum correlation between the social psychological domains of the OES and general health domains of SF36) and −0.84 (the maximum correlation between the elbow function domain of the OES and DASH). All correlations are statistically significant. Cronbach’s alpha coefficients are 0.89, 0.90 and 0.84, respectively. Each item is scored from 0 to 4, with higher scores denoting greater severity [6].

#### Short Form 36

SF36 domains were used to test the convergent and divergent validity of the Turkish version of the OES. A 36-item short form, constructed to survey health status in the medical field, was developed by Ware and Sherbourne [10]. The SF36 includes one multi-item scale that assesses eight health concepts: (1) limitations in physical activities because of health problems, (2) limitations in social activities because of physical or emotional problems, (3) limitations in usual role activities because of physical health problems, (4) bodily pain, (5) general mental health (psychological distress and well-being), (6) limitations in usual role activities because of emotional problems, (7) vitality (energy and fatigue) and (8) general health perceptions. Pınar et al. [11] adapted the SF36 to the Turkish culture.

### Procedure

While adapting the original version of the OES to the Turkish culture, the following steps were tracked according to the Isis Outcomes Translation and Linguistic Validation Process http://isis-innovation.com/outcome-measures/the-oxford-elbow-score-oes/:

1. Conceptual definition: Each item of the original version of the OES was examined to ensure that they would be equivalent in the Turkish version. When it was ensured that all items had the same conceptual meanings in the Turkish culture, we proceeded to the forward translation.
2. Forward translation: Two translators independently produced forward translations. One of the translators had a clinical and medical background in elbow luxation and fractions. The other translator was a linguistics expert and offered a translation that reflected the language used by the common population. Both translators were experienced in medical translations.
3. Back translation: Next, two additional translators independently back translated the translated version of the OES into English. Those translators did not see the original English wording of the OES. One of those translators had a clinical and medical background and was knowledgeable about elbow problems. The other translator was a native English speaker. Both translators were experienced in medical translations.
4. Back translation review: A group of experts, including the researchers, reviewed the back translations against the source version to highlight any discrepancies in meaning or terminology used.
5. Pilot testing: The translated OES was pilot tested with five patients who suffered from elbow problems. Each patient was asked during the face-to-face interview to comment on any wording that was difficult to understand and suggest alternative wording/phrasings for any difficult-to-understand items, etc.
6. Pilot testing review: A group of experts, including the researchers, reviewed the comments on the pilot testing report to highlight any discrepancies in meaning or terminology used. As a result, the final form of the OES was developed.
7. Psychometric testing/validation: At this step, the draft form of the OES was administered to 82 patients. Then, some statistical tests were calculated to assess the validity and reliability of the OES.

### Analysis of data/statistical analyses

In this section, validity and reliability studies are presented.

#### Construct validity

To define construct validity of the scale, confirmatory factor analyses were calculated. The Kaiser-Meyer-Olkin Measure of Sampling Adequacy (KMO) and Bartlett’s Test of Sphericity were calculated to test if the data was suitable for the factor analysis. The KMO tests whether the partial correlations among variables are small. In other words, the calculations represent the ratio of the squared correlation between variables to the squared partial correlation between variables [12]. Bartlett’s test is another indication of the strength of the relationship among variables. Bartlett’s test examines if the population matrix resembles the identity matrix.

For the confirmatory factor analysis, the model fit was assessed using the ratio of chi square to degrees of freedom (*χ*^2^/sd), a goodness of fit index (GFI), an adjusted goodness of fit index (AGFI), a comparative fit index (CFI), a non-normed fit index (NNFI), a root mean square error of approximation (RMSEA), and a standard root mean square residual (SRMR). Table 2 presents the cut-off points for these fit indices.

**Table 2:** Fit indices and cut-off points [14, 15]

Fit indices	Cut-off points
*χ* ^2^/sd	≤2.5 excellent fit, ≤5 mediocre fit
GFI-AGFI-CFI-NNFI	≥0.95 excellent fit, ≥0.90 good fit
SRMR-RMSEA	≤0.05 excellent fit, ≤0.08 good fit, ≤0.010 poor fit

#### Item discrimination

The discrimination levels of the Turkish version of the OES items were computed to investigate whether there was a significant difference between the total scale scores and the factor scores of the participants from the upper and lower 27 %. Item discrimination was calculated for the domains of the OES. An independent sample *t* test was calculated to determine if there was a significant difference between the scores of the participants from the upper and lower groups.

#### Internal consistency

Cronbach’s alpha internal consistency coefficient was calculated for the three sub-factors of the OES (pain, elbow function and social/psychological) in order to explore if the Turkish version of the OES measuring the same general construct produced a similar score.

#### Test–retest reliability (reproducibility)

To assess the test–retest reliability of the Turkish version of the OES, 40 patients, at the first assessment, were asked to complete and return a second questionnaire 24 h after the first. A Pearson correlation coefficient was calculated to explore the relationship between the two assessments. Moreover, a repeated measure *t* test was calculated if there was a change in the distribution of scores between the two tests.

#### Convergent validity

A Pearson correlation coefficient and Spearman’s correlation coefficient were calculated to assess the relationship between the Turkish version of the OES and the SF36 domains. A Pearson correlation coefficient requires all variable scores to be continuous and normally distributed. Thus, at first, the normality assumption was tested for all variables.

## Results

### Construct validity

According to the results of the KMO test, the KMO value was 0.829. Kaiser [13] mentioned that 0.8 and 0.9 are great values. On the other hand, the results of Bartlett’s test were found to be highly significant (*p* < 0.0001). Considering the results of the KMO and Bartlett’s test, the data were considered suitable for factor analysis.

The original form of the OES has 12 items as part of the three factors. They are pain (items 7, 8, 11 and 12), elbow function (items 1–4) and social/psychological (items 5, 6, 9 and 10). A confirmatory factor analysis was conducted to test the three-factor structure of the scale that was identified in the original form of the OES. Table 3 presents observed variables, latent variables and abbreviations for latent variables.

**Table 3:** Latent and observed variables

Latent variables	Abbreviations for latent variables	Observed variables
Pain	P	R7, R8, R12, R11
Elbow function	E	R4, R3, R2, R1
Social/psychological	S	R10, R6, R5, R9

**Fig. 1:**
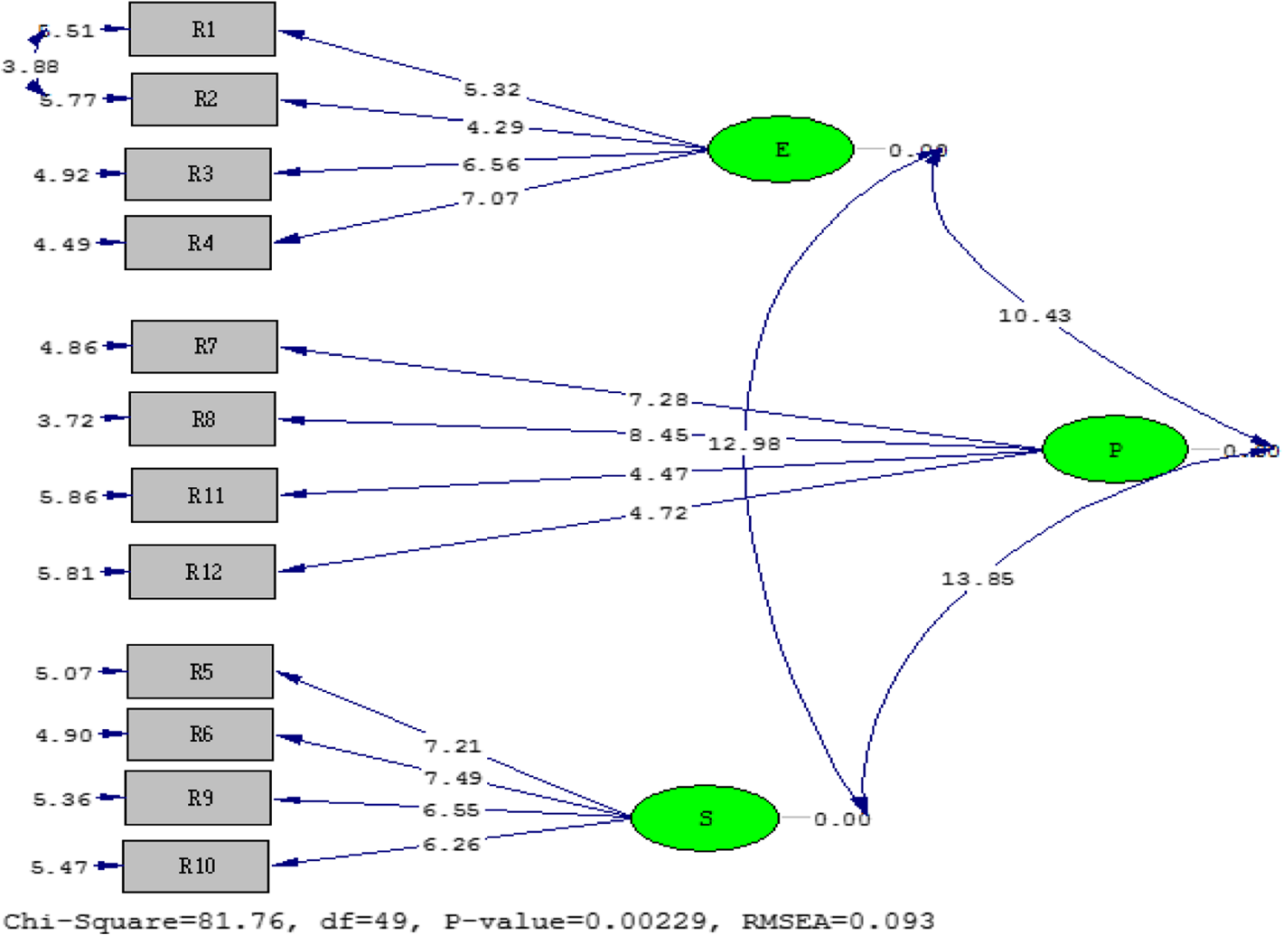
*T* values for the relationship between latent and observed variables

**Fig. 2:**
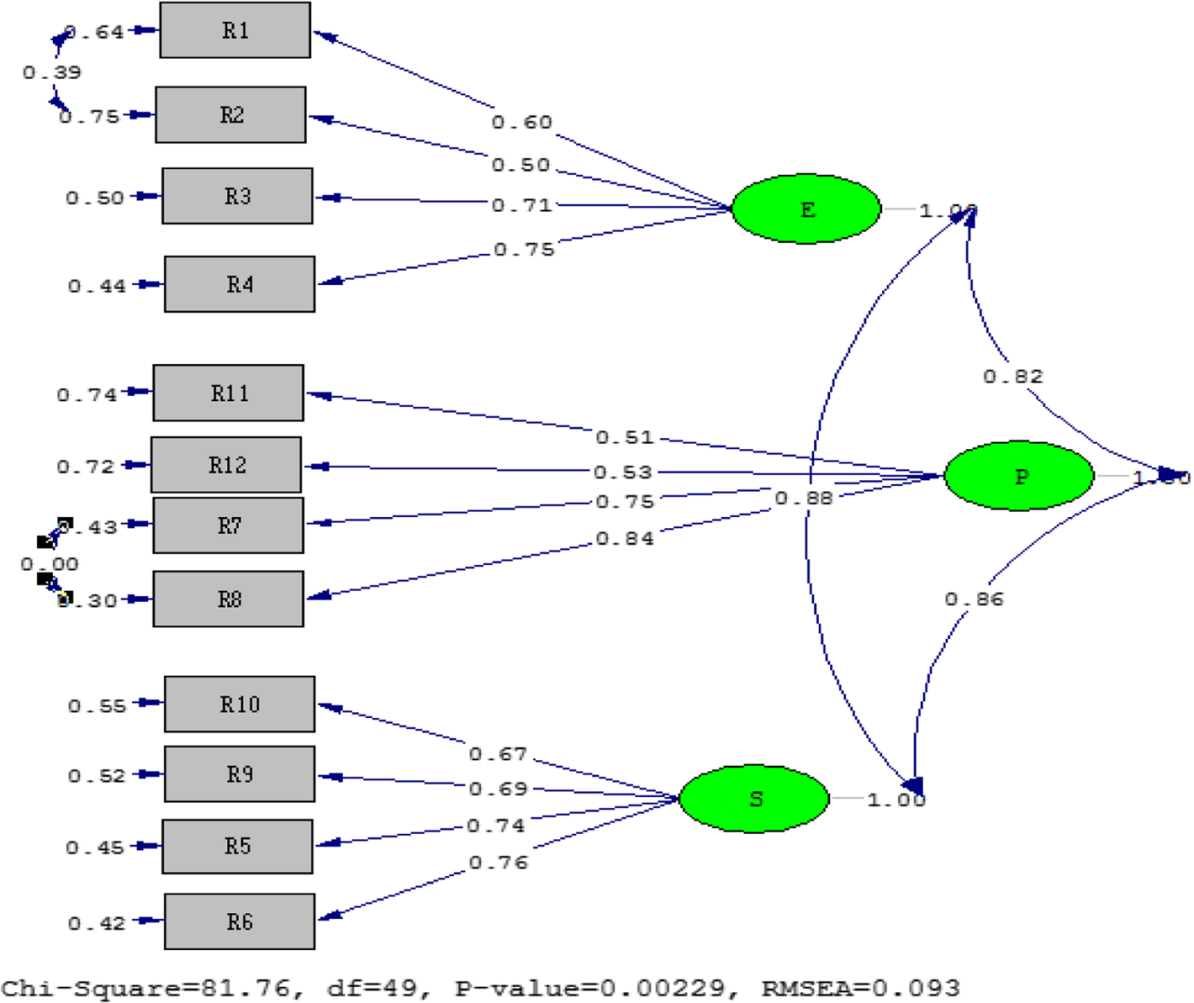
Standardised coefficients for the relationship between latent and observed variables

The confirmatory factor analysis indicated that the *t* values for the relationship between latent and observed variables were above the critical ratio (2.99) and were statistically significant at the 0.01 level. Figures 1 and 2 demonstrate the *t* values and standardised coefficients, respectively.

The modification indices showed that there was a significant decrease in the *χ*^2^ value when items 1–2 and 7–8 were modified. Based on reviews and experts views, it was decided that the modifications had a theoretical basis. Following the modifications, the analysis was re-conducted and the ratio of *χ*^2^ to degrees of freedom was calculated as 81.76/49 = 1.67. This indicated an excellent fit [14]. The results of the other fit indices are as follows: NNFI 0.94, CFI 0.96, GFI 0.85, AGFI 0.76, RMSEA 0.09 and standardised RMR 0.07.

The results indicated that the CFI value was above 0.95 and indicated an excellent fit. On the other hand, the NNFI value indicated a good fit as it was above 0.90 [15, 16]. The standardised RMR value below 0.08 indicated a good fit. The RAMSEA value showed a poor fit as it was below 0.010. The GFI value indicated a mediocre fit. AGFI values, on the other hand, indicated a poor fit [14, 17].

The results showed that although the RMSEA and AGFI values were not at the expected level, the ratio of *χ*^2^ to degrees of freedom, the NNFI, CFI, GFI and standardised RMR values were at the expected level, indicating an excellent and good fit. When the results were examined in general, although the AGFI value was lower and the RMSEA value was a little bit higher than expected, the three-factor structure of the OES was considered confirmed.

### Reproducibility (test–retest reliability)

The results showed that there were positive and high correlations between the first and follow-up assessments (*r* = 0.89 *p* < 0.0001). Test–retest reliability coefficients for pain, elbow function and social/psychological dimensions of the Turkish version of the OES were, respectively, 0.88, 0.79 and 0.87, which are statistically significant at the level of 0.001.

Moreover, the results of the repeated measure *t* test showed that the mean scores of the first and second assessments are not significantly different from zero for both the three sub-dimensions and the whole of the scale (the whole scale: *t*_(39)_ = 2.089 *p* > 0.001; pain: *t*_(160)_ = −0.863, *p* > 0.001; elbow function: *t*_(160)_ = −1.772, *p* > 0.001; social/psychological: *t*_(160)_ = −0.665, *p* > 0.001).

### Internal consistency

The internal consistency of the questionnaire for the whole scale was 0.91. When looking at the results, there was a high internal consistency. All items correlated with the total score of more than 0.5, except for item 11. In each case, Cronbach’s alpha value remained higher than 0.89. For the pain, elbow function and social/psychological sub-factors, Cronbach’s alpha values were, respectively, 0.76, 0.79 and 0.83.

### Convergent validity

Table 4 presents the correlation between SF36 domains and the Turkish version of the OES domains.

**Table 4:** Correlation between SF36 and OES domains

SF36	Turkish OES version total score	Turkish OES pain dimension	Turkish OES elbow function dimension	Turkish OES social/psychological dimension
Physical functioning	0.48**	0.43**	−0.45**	0.38**
Social functioning	0.47**	0.50**	−0.38**	0.39**
Pain	0.73**	0.67**	−0.64**	−0.63**
Role physical	0.54**	0.50**	−0.47**	0.47**
Role emotional	0.25*	0.25*	0.18	0.24*
Vitality	0.16	0.24	0.10	0.10
Mental health	0.27*	0.29**	−0.23*	0.22*
General health	0.01	−0.12	0.13	−0.01

*p < 0.05; **p < 0.01

All variables were normally distributed, except for the physical functioning dimension of SF36. Thus, Spearman’s correlation coefficient was calculated to test the relationship between the OES domains and physical functioning. The results show that the Turkish OES version and its dimensions have moderate and significant correlations with the domains of the SF36 in general. However, there was no significant correlation between the Turkish OES version and the general health domains of the SF36 (Table 4).

### Item discrimination

The test results indicated that the mean of each item on the three domains of the OES was higher for the upper 27 %, and this difference was significant at the 0.01 level. This result showed that the items of the OES could discriminate between the participants in the lower and upper 27 %; in other words, the discrimination level of the Turkish version of the OES items is high.

## Discussion

The results of the statistical analysis show that the Turkish version of the OES is valid and reliable. The original form of the OES has 12 items as part of the three factors. They are pain (items 7, 8, 11 and 12), elbow function (items 1–4) and social/psychological (items 5, 6, 9 and 10). In our study, a confirmatory factor analysis (CFA) was used to confirm the three-factor structure of the OES. In the CFA, most of the fit indices were at the expected level, except for the AGFI and RMSEA values. Although those values were a little higher than expected, in general, the three-factor structure of the OES was confirmed.

The findings of our study also showed that the Turkish version of the OES was reproducible. The correlation between the pre- and post-tests was high (*r* = 0.89). On the other hand, there was no significant difference between the means of the pre- and post-tests. Those results were consistent with the values in the original version of the OES [6]. Moreover, in our study, we calculated reproducibility for each domain of the OES separately. In addition to the total score of the OES, the scores obtained from each domain were also reproducible (*r* = pain 0.88, elbow function 0.79, social/psychological 0.87; *p* < 0.01).

The results of our study showed that the internal consistency of the Turkish version of the OES was high (Cronbach’s alpha = 0.91), consistent with the original version of the OES (0.91). Furthermore, in our study, Cronbach’s alpha value was calculated for each domain independently. The values were 0.76, 0.79 and 0.83 for the pain, elbow function and social/psychological domains of the OES, respectively. Because there is a correlation between the number of items and the reliability of a test, Cronbach’s alpha value is lower in domains of the OES in comparison to the total score of the OES.

The convergent validity results also showed that the Turkish OES version and its dimensions have moderate and significant correlations with domains of the SF36 in general. However, there was an insignificant correlation between the Turkish OES version and the general health and vitality domains of the SF36. In the original development study of the OES, however, the lowest correlation was found between the OES domains and the general health and vitality domains of the SF36 [6]. In the original version of the OES, vitality had a low and insignificant correlation (in the first assessment) with the OES domains. In our study, we could not find a significant correlation between those variables either. In general, our findings demonstrated that there is a moderate correlation between the domains of the Turkish version of the OES and SF36. However, those correlation coefficients are relatively low in comparison with the coefficients in the original development study of the OES.

The findings of our study showed that the mean of each item on three domains of the OES was higher for the upper 27 %. This proves that items in the Turkish version of the OES discriminate between the patients who have significant elbow problems (upper 27 %) and who have more minor elbow problems (lower 27 %). This proves that Turkish version of OES could be used by clinicians to determine severity of the elbow problems effectively.

## Conclusions

As a result, the Turkish version of the OES:

- Has high internal consistency
- Is reproducible
- Can discriminate the patients with significant and minor elbow problems
- Has high construct validity
- Has moderate correlation with SF36 domains

It is recommended that the Turkish version of OES could be used to assess the patients’ functional status for research purposes. It could also be used to identify improvements in patients with elbow problems in clinics.

## Abbreviations

OES: Oxford Elbow Score
MEPS: Mayo Elbow Performance Score
DASH: Disabilities of the Arm, Shoulder and Hand outcome measure
KMO: The Kaiser-Meyer-Olkin Measure of Sampling Adequacy
SF36: Short Form 36
*χ*^2^/sd: The ratio of chi square to degrees of freedom
GFI: Goodness of fit index
AGFI: Adjusted goodness of fit index
CFI: Comparative fit index
NNFI: Non-normed fit index
RMSEA: Root mean square error of approximation
SRMR: Standard root mean square residual
CFA: Confirmatory factor analysis

## References

[1] Fitzpatrick R, Fletcher A, Gore S, Jones D, Spiegelhalter D, Cox D (1992). Quality of life measures in health care. I: applications and issues in assessment. BMJ.

[2] Testa MA, Simonson DC (1996). Assessment of quality-of-life outcomes. N Engl J Med.

[3] Patrick D, Guyatt G, Acquadro C, Higgens J, Green S (2008). Patient-reported outcomes. Cochrane handbook for systematic reviews of interventions.

[4] Longo UG, Franceschi F, Loppini M, Maffulli N, Denaro V (2008). Rating systems for evaluation of the elbow. Br Med Bull.

[5] Reininga IHF, Moumni ME, Eygendaal D, The B (2013). Elbow-specific clinical rating systems: extent of established validity, reliability, and responsiveness. J Shoulder Elb Surg.

[6] Dawons J, Doll H, Boller J, Fitzpatrick R, Little C, Rees J, Jenkinson C, Carr AJ (2008). The development and validation of a patient reported questionnaire to assess outcomes of elbow surgery. J Bone Joint Surg.

[7] Haan J, Goei H, Schep NW, Tuinebreijer WE, Patka P, Hartog D (2011). The reliability, validity and responsiveness of the Dutch version of the Oxford elbow score. J Orthop Surg Res.

[8] Plaschke HC, Jørgensen A, Thillemann TM, Brorson S, Olsen BS (2013). Validation of the Danish version of the Oxford Elbow Score. Danish Med J.

[9] Barrett P, Kline P (1981). The observation to variable ratio in factor analysis. J Pers Group Behav.

[10] Ware JE, Sherbourne DC (1992). The MOS 36 item short form health survey (SF 36). Med Care.

[11] Pınar R (1995). Sağlık araştırmalarında yeni bir kavram: Yaşam kalitesi, bir yaşam kalitesi ölçeğinin kronik hastalarda geçerlik ve güvenirliğinin sınanması. Hemşirelik Bülteni.

[12] Field A (2005). Discovering statistics using SPSS.

[13] Kaiser HF (1974). An index of factorial simplicity. Pstchometrika.

[14] Tabachnick BG, Fidell LS (2001). Using multivariate statistics.

[15] Sümer N (2000). Yapısal eşitlik modelleri. Türk Psikoloji Yazıları.

[16] Hooper D, Coughan J, Mullen M (2008). Structural equation modeling: guidelines for determining model fit. Electron J Bus Res Methods.

[17] Brown TA. Confirmatory factor analysis for applied research. New York: Guilford Publications; 2015.

